# Supplementary material for: Monitoring the Spread of Grapevine Viruses in Vineyards of Contrasting Agronomic Practices: A Metagenomic Investigation
Source: Biology (Basel). 2023 Sep 25;12(10):1279. doi: 10.3390/biology12101279 (PMC10604868; doi:10.3390/biology12101279)
Supplement: Supplementary file 1 [file biology-12-01279-s001.zip › biology-2544880-supplementary.pdf]

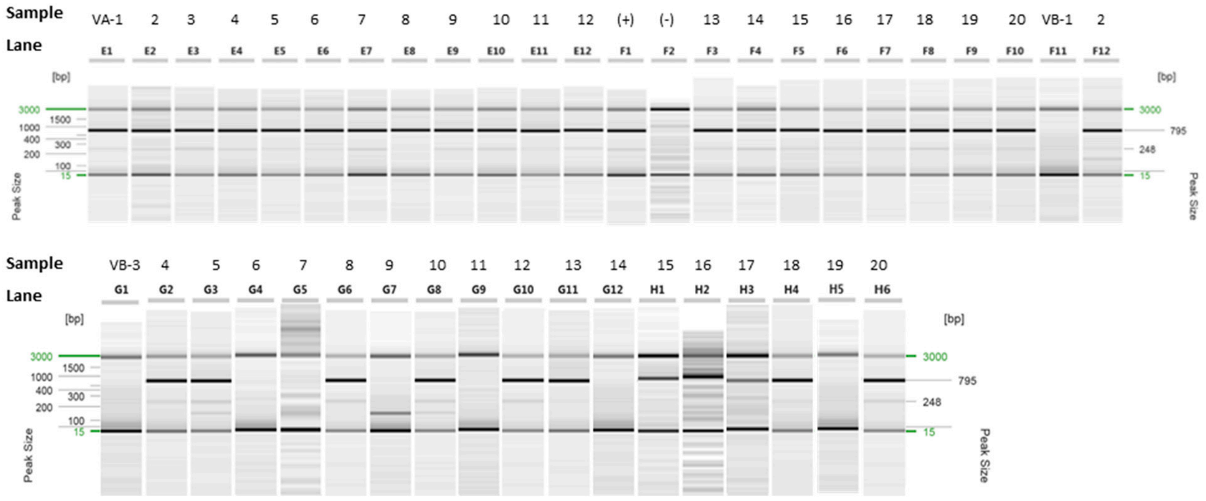

Figure S1: PCR test for GPGV of the forty commercial vine samples. Lane E1 to E12 and F3 to F10 are samples from the organic vineyard A (VA-1 to VA-20). Lane F11 to H6 are samples from the conventional vineyard B (VB-1 to VB-20). Lane F1 and F2 are positive and negative controls for GPGV, respectively. Visualization was carried out by the QiAxcel gel electrophoresis system.

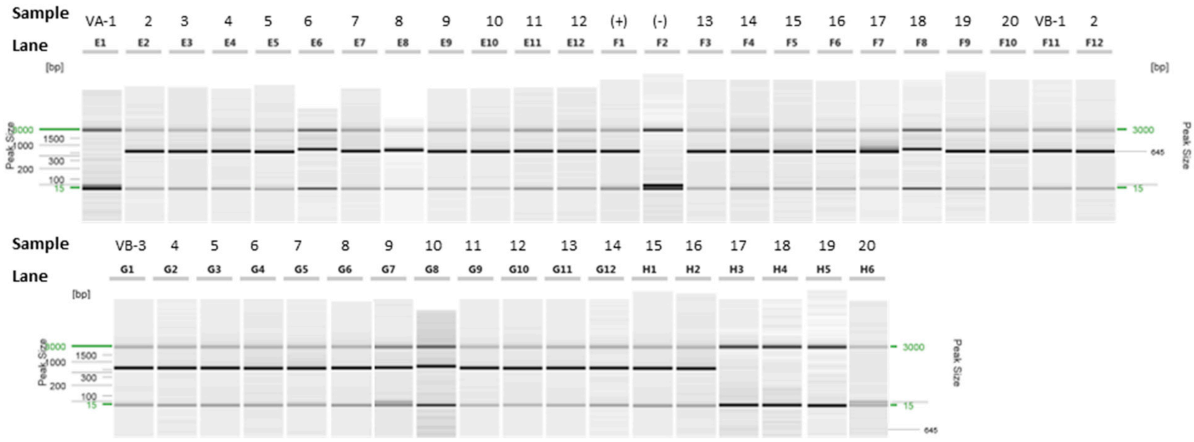

Figure S2: PCR test for GRBV of the forty commercial vine samples. Lane E1 to E12 and F3 to F10 are samples from the organic vineyard A (VA-1 to VA-20). Lane F11 to H6 are samples from the conventional vineyard B (VB1-VB-20). Lane F1 and F2 are positive and negative controls for GRBV, respectively. Visualization was carried out by the QiAxcel gel electrophoresis system.

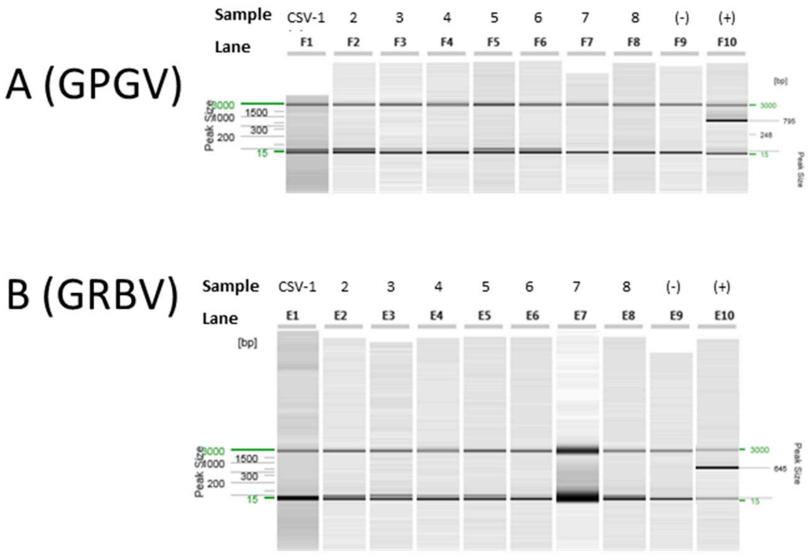

Figure S3: PCR test for GPGV (A, top) and GRBV (B, bottom) of the eight composite samples from sentinel vines. Lane F1 to F8 and E1 to E8 are the composite samples from the sentinel vines (CSV-1 to CSV-8). Lane F9 and F10 are the negative and positive control for GPGV repsectively. Lane E9 and E10 are the negative and positive control for GRBV respectively. Visualization was carried out by the QiAxcel gel electrophoresis system.

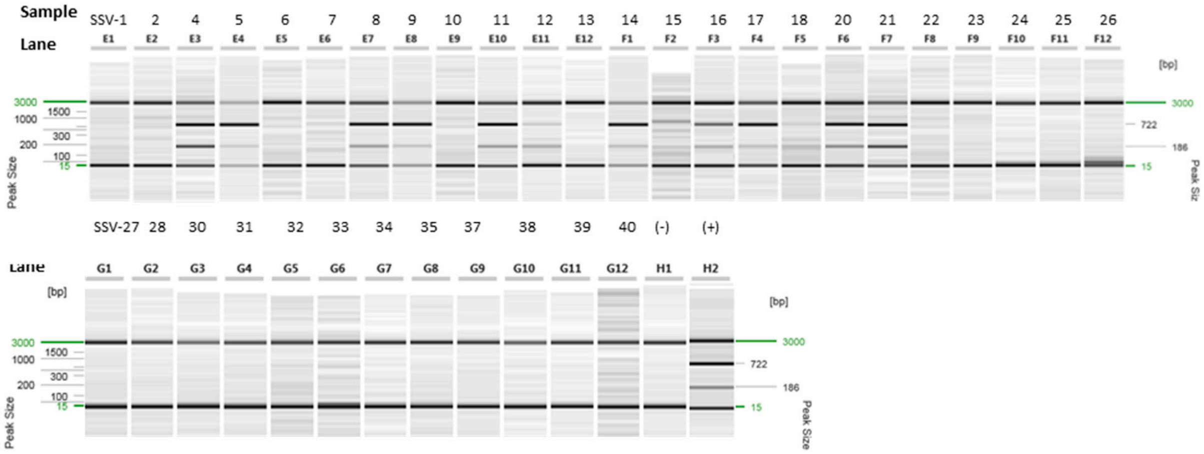

Figure S4: PCR test for GPGV of the 37 survived sentinel vine samples. Lane E1 to F6 are samples from the organic vineyard A (SSV-1 to SSV-20). Lane F7 to G12 are samples from the conventional vineyard B (SSV-21 to SSV-40). Lane H1 and H2 are positive and negative controls for GPGV, respectively. Visualization was carried out by the QiAxcel gel electrophoresis system.

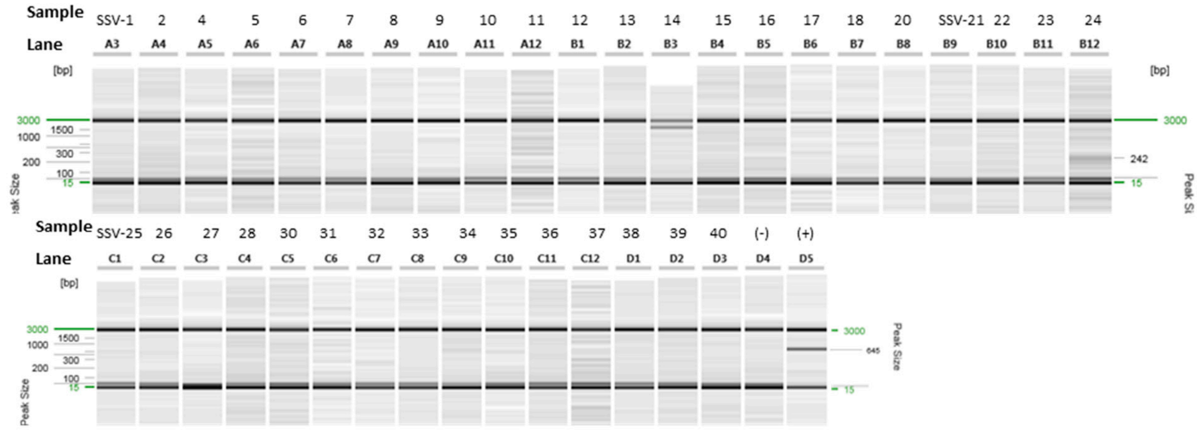

Figure S5: PCR test for GRBV of the 37 survived sentinel vine samples. Lane A3 to B8 are samples from the organic vineyard A (SSV-1 to SSV20). Lane B9 to D3 are samples from the conventional vineyard B (SSV-21 to SSV-40). Lane D4 and D5 are positive and negative controls for GRBV, respectively. Visualization was carried out by the QiAxcel gel electrophoresis system.
